# Discovery of neddylation E2s inhibitors with therapeutic activity

**DOI:** 10.1038/s41389-023-00490-2

**Published:** 2023-09-16

**Authors:** MAA Mamun, Ying Liu, Yin-Ping Geng, Yi-Chao Zheng, Ya Gao, Jian-Gang Sun, Long-Fei Zhao, Li-Juan Zhao, Hong-Min Liu

**Affiliations:** 1 Key Laboratory of Advanced Drug Preparation Technologies, Ministry of Education, China; State Key Laboratory of Esophageal Cancer Prevention & Treatment; Key Laboratory of Henan Province for Drug Quality and Evaluation; Institute of Drug Discovery and Development; School of Pharmaceutical Sciences, Zhengzhou University, 100 Kexue Avenue, Zhengzhou, 450001 China; 2 Henan Engineering Research Center for Application & Translation of Precision Clinical Pharmacy; Department of Pharmacy, the First Affiliated Hospital of Zhengzhou University, Zhengzhou, 450052 China; 3 Department of Gastrointestinal Surgery, the First Affiliated Hospital of Zhengzhou University, Zhengzhou, Henan 450052 China; 4 State Key Laboratory of Esophageal Cancer Prevention & Treatment, Academy of Medical Sciences, Zhengzhou University, Zhengzhou, 450052 China

**Keywords:** Cancer, Molecular biology

## Abstract

Neddylation is the writing of monomers or polymers of neural precursor cells expressed developmentally down-regulated 8 (NEDD8) to substrate. For neddylation to occur, three enzymes are required: activators (E1), conjugators (E2), and ligators (E3). However, the central role is played by the ubiquitin-conjugating enzymes E2M (UBE2M) and E2F (UBE2F), which are part of the E2 enzyme family. Recent understanding of the structure and mechanism of these two proteins provides insight into their physiological effects on apoptosis, cell cycle arrest and genome stability. To treat cancer, it is therefore appealing to develop novel inhibitors against UBE2M or UBE2F interactions with either E1 or E3. In this evaluation, we summarized the existing understanding of E2 interaction with E1 and E3 and reviewed the prospective of using neddylation E2 as a pharmacological target for evolving new anti-cancer remedies.

## Introduction

**Fig. 1:**
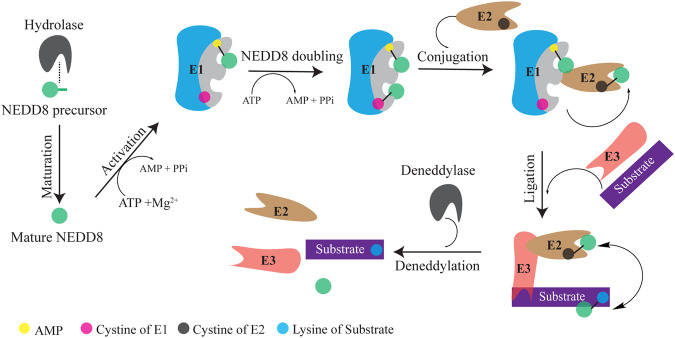
Canonical neddylation pathway. Precursor NEDD8 with hydrolase is the first process before NEDD8 reacts with NAE. By actions of ATP and Mg^2+^, NAE is first loaded with one NEDD8 molecule. Following the same procedure, the second NEDD8 is attached with NAE. Double NEDD8 loaded NAE allows conjugating enzyme E2 binding and transferring NEDD8 from NAE’s active cysteine to E2’s cysteine. Next, by coordinating with E3 ligase, NEDD8 moves to the target protein. Finally, through the deneddylation process, NEDD8 is removed from the substrates.

The neddylation pathway is a vital post-translational modification mechanism that plays a crucial role in several biological functions, including protein homeostasis, conformational change, and movement [1]. The process of neddylation is similar to that of ubiquitination. First, deneddylase 1 (DEN1) or ubiquitin C-terminal hydrolase domain 3 (UCH-L3) cleave the five amino-acid residues at the C-terminal end of the NEDD8 precursor, transforming it into mature NEDD8 (Fig. 1) [2, 3]. Once E1 recognizes the mature NEDD8, it initiates the neddylation process by utilizing the magnesium ion Mg^2+^ and adenosine triphosphate (ATP). To initiate conformational shifts in E1, which facilitate its binding to E2 and the subsequent transfer of NEDD8 to E2, two molecules of NEDD8 must initially bind to E1 [4]. After docking of E1’s adenylation domain with E2’s N-terminus, E1 releases the NEDD8 to E2 [5, 6]. In the final step, E2 works together with E3 ligases to transfer NEDD8 to the lysine residue of target molecules, which then activates downstream signaling pathways [7]. The NEDD8 modification is a reversible process carried out by deneddylase enzymes, including COP9 signalosomes (CSN), NEDD8-specific proteases (NEDP1), DEN1, and Ubiquitin carboxyl-terminal hydrolase 21, which remove NEDD8 from substrates [8–11]. While the classical neddylation pathway typically involves the addition of a single NEDD8 molecule to substrates, recent evidence suggests that polyneddylation can also occur and influence the function of substrates [12]. Several substrates of the neddylation pathway have been identified, including proteins in the CULLIN family, p53, the epidermal growth factor receptor (EGFR), E2F transcription factor 1 (E2F1), mouse double minute 2 (MDM2), and β-catenin [13, 14]. Currently, the neddylation pathway is known to be regulated by a single E1 enzyme or NEDD8 activating enzyme (NAE), two E2 enzymes (UBE2M/UBE2F), and over ten E3 enzymes [13].

The first E2 enzyme of the neddylation pathway to be discovered was the yeast-derived RUB1 conjugating enzyme (UBC12) [15]. Later, the human form of UBC12, known as UBE2M, was identified [5]. The second NEDD8 conjugating enzyme in humans, encoded by the UBE2F gene, was discovered in 2009 [16]. Initially thought to be mere “NEDD8 carriers” with limited functionality, modern research has revealed that E2 enzymes play essential roles in various physiological activities. UBE2M is overexpressed in several cancer types [17–20]. Normal physiological conditions rely on UBE2M for autophagy regulation, cell proliferation, stress granule assembly, and more [21–23]. Additionally, UBE2M utilizes multiple CULLIN ring ligases (CRLs) to ensure DNA damage repair and maintain genetic stability. Decreased expression of UBE2M results in the accumulation of CRL substrates chromatin licensing and DNA replication factor 1 (CDT1) and origin recognition complex subunit 1, which increases sensitivity to DNA-damaging agents [19, 24]. Cells expressing a reduced amount of UBE2M are sensitive to ionizing radiation and survive less well after nonhomologous end joining (NHEJ) [25]. Moreover, UBE2M depletion increases cancer cell sensitivity towards niraparib, fluorouracil, and oxaliplatin (chemotherapeutic agents) [26, 27]. Inhibition of UBE2M through siRNA treatment decreases cancer cell viability and clonogenic survival and inhibits tumor growth in vivo.

Meanwhile, overexpression of UBE2F in non-small cell lung cancer patients is associated with poor prognosis. In vitro and in vivo studies have demonstrated that UBE2F overexpression promotes lung cancer formation, whereas UBE2F knockdown impedes tumor growth [28]. Earlier studies showed that UBE2F could bind NEDD8 to CULLIN5, but recent studies have demonstrated that it can also interact with CULLIN1 [16, 29]. Once CULLIN5 is modified by UBE2F-mediated NEDD8, it leads to the formation of CRL5 and regulates the degradation of the pro-apoptotic protein NOXA [28]. If CULLIN5 neddylation is blocked, CRL5 is inhibited, and NOXA builds up, resulting in apoptosis. Therefore, UBE2F suppression is a viable target for anti-tumor therapy, as it promotes apoptosis and inhibits cancer cell development.

A potent inhibitor targeting the NAE, MLN4924, is currently undergoing phase III clinical trials [13]. In addition to inhibiting E1 of NEDD8, MLN4924 also inhibits E1 of ubiquitination and sumoylation, but with less potency [30]. Therefore, inhibiting ubiquitination and sumoylation along with neddylation can potentially result in adverse effects of MLN4924. Furthermore, mutations in the ATP-binding pocket of UBA3 can limit the response to MLN4924 and reduce its clinical application [31]. Hence, alternative targets in the neddylation pathway are urgently needed. In this case, UBE2M and UBE2F emerge as strong candidates for anticancer drug discovery. It is crucial to target specific E2 or E2–E3 interactions to selectively inhibit the neddylation of specific CULLIN or other substrates since E1 inhibition can have a wide range of unfavorable effects. For instance, HIF1 accumulation can increase cancer cell survival in hypoxic conditions due to CRL2 neddylation inhibition. Nuclear factor erythroid 2-related factor 2 (NRF2) accumulation via CRL3 inhibition can contribute to cancer growth, resistance to chemotherapy and radiation, and poor prognosis for patients [32, 33]. This review incorporates the most recent information regarding E2s of neddylation, their interactions with E1 and E3, possible consequences of E2 inhibition, and methods for identifying E2 targeting inhibitors.

## Structural signature of UBE2M and UBE2F and its application on inhibitor development

**Fig. 2:**
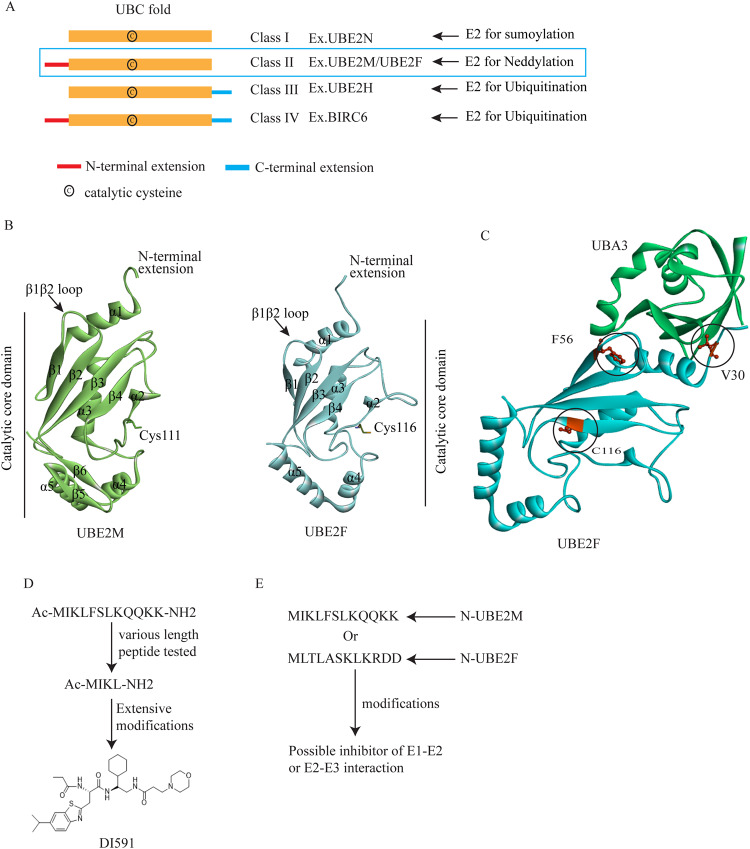
Structural features of UBE2M and UBE2F. **A** Classification of E2 conjugating enzyme based on N and C terminal extensions. **B** The crystal structure of UBE2M in green (PDB: 1Y8X) and UBE2F in the sky (PDB: 3FN1). **C** Virtually identified V30, F56, and C116 pockets of UBE2F for inhibitor binding. **D** Principle of developing DI-591 and (**E**) Proposed idea of developing E2 targeting inhibitor.

E2 family proteins typically have a well-preserved 150–200 amino acid-long ubiquitin-conjugating catalytic (UBC) domain [34]. This domain shares 35% similarity among different members of the E2 protein family, and it provides a platform for E1, E3s, and activated ubiquitin-like (UBL) proteins to bind [34]. The catalytic cysteine is embedded within the UBC domain. In addition to the UBC domain, E2 classification is also influenced by the existence of extra amino acid chains in the N or C terminal region of the UBC domain (Fig. 2A) [35]. Based on this arrangement, four classes of E2s have been identified. Class I consist only of the catalytic UBC domain, such as SUMO conjugating enzyme UBE2N. Class III contains a C-terminal extension (e.g., Ubiquitin-conjugating enzyme E2H, UBE2H), while class IV is composed of both N- and C-terminal extensions (e.g., Baculoviral IAP repeat-containing protein 6, BIRC6) [35]. Class II contains only an extended N-terminal end. UBE2M and UBE2F belong to this class of proteins with 26 residues of N-terminal extension, which distinguishes them from other UBL E2s [6, 16]. The effectiveness of NEDD8 E2s at transferring NEDD8 to substrates is significantly reduced when the N-terminus is deleted. Mutant UBE2M does not promote proliferation, although wild-type and malformed UBE2M proteins are expressed at similar levels [36]. UBE2M and UBE2F’s core domains adopt the common E2 core domain folding pattern [37]. The catalytic cysteine of UBE2M is located at position 111, while that of UBE2F is at position 116 [16]. Although both proteins have the same number of α helices, UBE2M has two additional anti-parallel β sheets at the C-terminal end (Fig. 2B). The residues present in the β1/β2 loop and α1 helices are responsible for the interaction with the neddylation fold domain of NAE [16]. An in-silico analysis of the interaction between UBE2F and UBA3 has identified two binding sites that can be targeted for creating E2-E1 interaction inhibitors (Fig. 2C) [38]. Additionally, drug screening was conducted on the catalytic cysteine located at position 116 of UBE2F. Similar analyses can be used for UBE2M, which could help in developing new neddylation inhibitors. The N-terminal end of UBE2M is known to undergo acetylation, connecting with the E3 ligase defective in CULLIN neddylation 1 (DCN1) and altering the CRL3. Haibin Zhou and colleagues developed DI591, a high-affinity peptidomimetic small molecule that mimics the acetylated N-terminal end of UBE2M (Fig. 2D) [39]. Similarly, an acetylation at the N-terminus of UBE2F has been found to be responsible for its interaction with DCN3 [40]. Therefore, targeting the acetylated N-terminal end of UBE2F could lead to the development of novel and potent neddylation inhibitors similar to DI-591 (Fig. 2E).

## E2-based protein-protein interactions and their downstream effects on cancer

There is substantial evidence indicating that both UBE2M and UBE2F are crucial in controlling cancer cell growth, immune function, and survival. UBE2M is involved in the neddylation of various CULLIN and non-CULLIN proteins, making it a popular research subject. However, more investigation is needed on UBE2F. Recently, several reviews have provided detailed overviews of the roles of both UBE2M and UBE2F in various cancers [1, 41]. While both E2s receive NEDD8 from a single E1, they need different E3 ligases that can either support cell survival or lead to cell death. In this section, we will provide a summary of recent findings related to the functions of both E2s and their interactions with E1 and E3.

### E2-E1 interaction

**Fig. 3:**
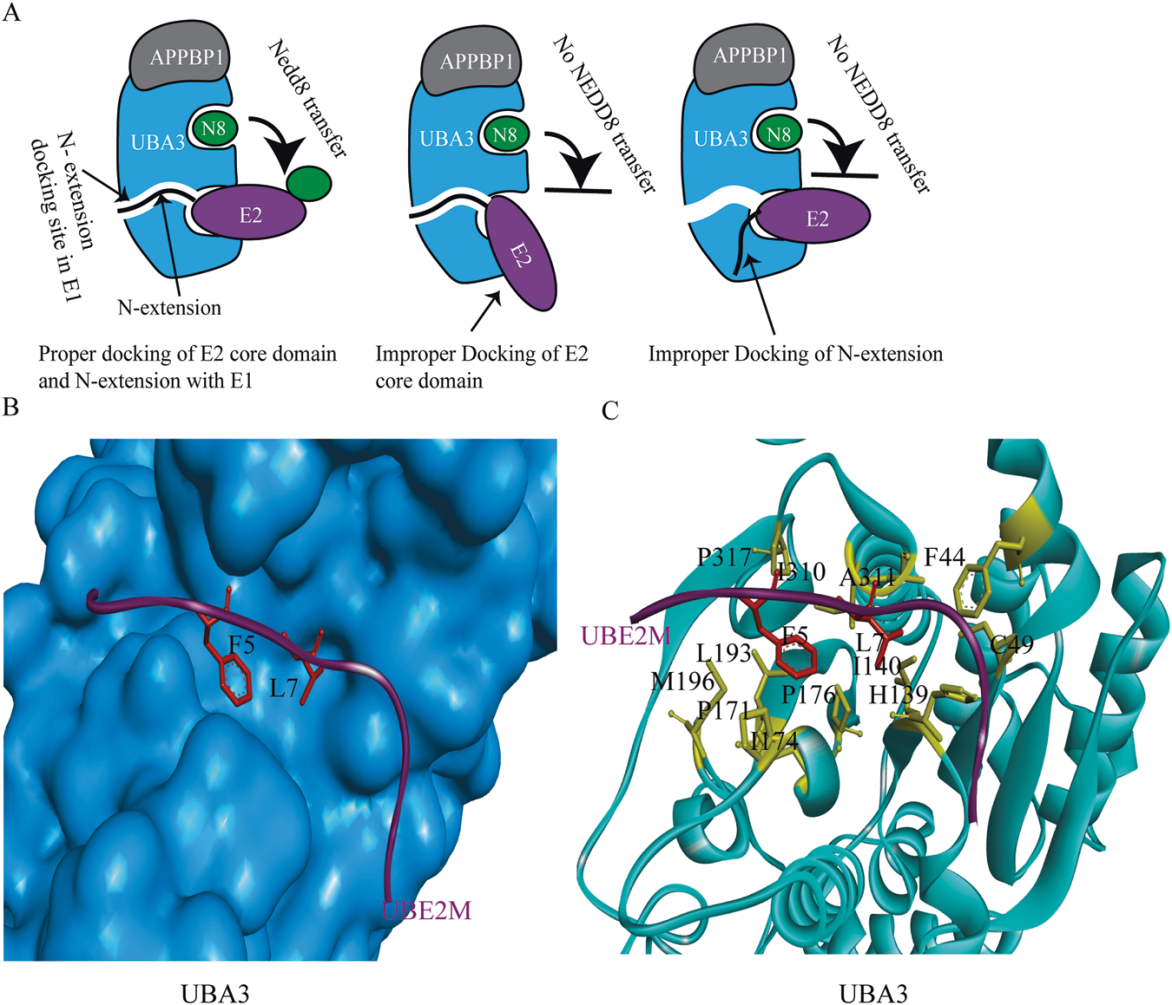
Structural analysis of E1-E2 binding pattern. **A** Interaction of E2 with E1. Appropriate interaction is necessary for NEDD8 transfer to E2. N-extension= N-terminal 26 residues of E2 (UBE2M). Improper docking of the E2 core domain or N-terminal extension prevented NEDD8 transfer from E1 to E2. **B,** **C** UBE2M’s N-terminal (purple) interaction with the hydrophobic pocket of UBA3 (cyan) and interacted amino acids, respectively (PDB:1TT5).

NAE is the only E1 enzyme involved in the neddylation process, which activates and transfers NEDD8 to either UBE2M or UBE2F. Analysis of the E2 structure revealed that the N-terminal extension of E2s is unique and necessary for docking in E1’s pocket [6]. However, further research is required to determine the situation when E1 selects between UBE2M or UBE2F. The E1 enzymes of neddylation consist of two heterodimeric subunits, amyloid precursor protein-binding protein 1, and UBL modifier activating enzyme 3 (UBA3) [42]. Proper interaction between the E2 enzymes and the UBA3 domain is crucial for the transfer of NEDD8 to E2 (Fig. 3A) [6, 36]. The N-terminal amino acids L4-F5-S6-L7 in UBE2M and M1-L2-T3-L4 in UBE2F bind to the docking groove of UBA3 [16], which follows an “HPR-HPR-AR-HPR” pattern (“HPR” representing hydrophobic residues, and “AR” representing any amino acid residue). Notably, hydrophobic residues F5 and L7 play a vital role in the proper docking of UBE2M’s N-terminal end with the large hydrophobic cavity of UBA3 (Fig. 3B, C) [36]. This unique property is exclusive to the E1-E2 neddylation interaction. Selective NAE-UBE2M or NAE-UBE2F interaction study is necessary to inhibit specific downstream neddylation. Therefore, targeting specific pockets in E1 or E2 that are involved in their interaction could lead to the development of new potential anti-cancer drugs.

### E2–E3 interactions

There are over 10 E3 ligases that interact with UBE2M or UBE2F to attach NEDD8 to specific substrates (Table 1). Of these, eight belong to the Ring finger domain-containing E3 class and they are RING-box protein 1 and 2 (RBX1 and RBX2), ring finger protein 111 and 168 (RNF111and RNF168), RNA polymerase II transcription factor B subunit 3 (TFB3), MDM2, casitas B lineage lymphoma (c-Cbl), and inhibitor of apoptosis (IAPs). Three E3s belong to the homologous to the E 6-AP carboxyl terminus (HECT) domain-containing E3 class and they are DCN1, itchy E3 ubiquitin ligase (ITCH), and smad ubiquitination regulatory factor 1 (SMURF1). The presence of HECT or RING domains distinguishes these two classes, which are crucial for their E3 ubiquitin ligase activity [43]. Tripartite motif-containing protein 40 (TRIM40) and F-box only protein 11 have also been identified as NEDD8 E3 ligases, however, their interaction with E2 conjugating enzyme was not reported yet [44, 45]. This section discusses how the interaction between E2 and E3 affects the neddylation of various types of substrates.

**Table 1:** E2 mediated different actions on cancer cells upon neddylation.

E2	E3	Downstream effects	Effects on cancer cell	References
UBE2M	RBX1	CRL1 activation	Increase cell proliferation and survivability	[46]
		CRL2 activation	Decrease cell propagation and angiogenesis	[32]
		CRL3 activation	Decrease cell survivability	[47]
		CRL4 activation	Increase DNA damage repair and inhibit apoptosis	[48]
	RNF111	H4 polyneddylation and RNF168 foci formation	Enhance DNA damage repair process	[49]
	RNF168	RNF168 auto-neddylation and H2A neddylation	Enhance DNA damage repair process	[50]
	MDM2	p53 neddylation	Inactivate p53	[51]
	IAP	Caspase neddylation	Caspase deactivation and apoptosis inhibition	[52]
	c-Cbl	c-Src degradation	Inhibits cell migration	[53]
		TβRII stabilization	Sensitizes leukemia cells to TGF-β	[54]
		EGFR sorting and degradation	Not reported	[55]
	TFB3	CRL3 activation in yeast	Not reported	[56]
	DCN1	CRL3 activation	Decrease cell survivability	[57]
	ITCH	ITCH auto-neddylation and JUNB neddylation	JUNB degradation	[58]
	SMURF1	SMURF1 auto-neddylation and stabilization	Increase tumor size, cancer cell proliferation and invasion	[59]
UBE2F	RBX2	CRL5 activation and NOXA degradation	Inhibit apoptosis	[16]

#### 3.2.1. Interactions with Ring type E3 ligases

##### 3.2.1.1. UBE2M–RBX1 interactions

RBX1 is a crucial component of the CRL1 complex, which is involved in the timely degradation of various substrates via ubiquitin ligation. In addition to its E3 ligase function for ubiquitin, RBX1 also interacts with UBE2M to facilitate the neddylation of CULLIN1-3, CULLIN4A, and CULLIN4B. This results in the formation of CRL1, CRL2, CRL3, CRL4A, and CRL4B, which promotes substrate ubiquitination and degradation [46]. RBX1’s structure consists of an N-terminal domain that binds to a substrate cullin’s C-terminal domain (CTD) and a C-terminal RING domain that attracts the UBE2M-NEDD8 intermediate. Upon neddylation, the RING domain stabilizes the UBE2M-NEDD8 in an active, closed conformation that can be nucleophilically attacked on the thioester link. To facilitate the transfer of NEDD8 to the substrate acceptor lysine residue, RBX1 brings the UBE2M-NEDD8 intermediate close to the bound substrate [47]. A recent study discovered that the UBE2M-RBX1 partnership is essential for regulatory T (Treg) cell fitness, indicating that neddylation maintains control of immunosuppressive function [46, 48]. Besides, by controlling various CRLs, RBX1-mediated neddylation controls the degradation of several critical substrates including p21, p27, CDT1, hypoxia-inducible factor 1-alpha (HIF1α), and NRF2, which are closely associated with cancer progression or inhibition.

*Cancer promoting functions (CRL1 and CRL4):* CULLIN 1 is a crucial part of the SCF (SKP1-CUL1-F-box protein) E3 ubiquitin ligase complex, which catalyzes the ubiquitination of various proteins for proteasomal degradation. Many proteins are degraded as a result of its activity, including transcription factors (Myc, IκBα, early growth response-1), cell cycle regulators (p21, p27, WEE1, CYCLINS D, and E), as well as apoptosis regulators (MCL-1, BimEL) [49]. CRL4A and CRL4B promoted the degradation of DNA damage-responsive protein CDT1 and cell cycle inhibitor p21 [50–53]. Besides, CRL4A promoted the IκB degradation to promote NF-κB activation which limits the apoptosis [54]. As UBE2M and RBX1 interaction is crucial for the assembly and activation of CRL1 and CRL4A/4B, inhibiting their formation could be a promising anticancer therapy. By targeting the inhibition of UBE2M and RBX1 interaction, it may be possible to develop new neddylation inhibitors with potential anticancer properties.

*Cancer inhibiting functions (CRL2 and CRL3):* CULLIN2 and CULLIN3 are the critical components of CRL2 and CRL3, respectively. When neddylation is inhibited in hypoxic conditions (favorable for tumor growth), CRL2 is deactivated, leading to an accumulation of HIF1α and increased stimulation of the HIF1 transcription factor [32, 55]. Activated HIF1 then promoted cell survivability, propagation, and angiogenesis by triggering various target genes responsible for regulating these processes [32]. This activation of HIF1 promotes the survival, proliferation, and angiogenesis of cells by inducing the expression of target genes that regulate these processes. Similarly, inactivated CRL3 results in the accumulation of NRF2, which has antioxidant properties that promote cell survival [33]. These findings show that the degradation of substrates by CRL2 and CRL3 limits cancer progression. Therefore, inhibiting the neddylation of CULLIN2 or CULLIN3 may not be beneficial for cancer treatment. Further research is needed to determine the specific conditions that result in the formation and activation of different CRLs.

##### UBE2F-RBX2 interactions

RBX2 is another NEDD8 E3 ligase in the ring protein family that activates CRL5 when interacting with UBE2F [56]. CRL5 regulates the ubiquitination and degradation of proteins such as Src kinase, the proapoptotic protein NOXA, and the tumor suppressor DEPTOR [57]. CRL5 uses the K11 linkage to control the polyubiquitination and proteasomal destruction of NOXA. Blocking the formation of CRL5 through UBE2F- and RBX2-mediated neddylation is crucial to inhibit NOXA degradation and increase cancer cell survival [28]. DEPTOR is a tumor suppressor that inhibits mTOR activity by directly binding to mTOR complexes [58]. DEPTOR degradation leads to mTOR activation, which promotes cell survival and proliferation while inhibiting autophagy. RBX2 and CUL5 work together to enhance DEPTOR ubiquitination and degradation in both in vitro and in vivo studies [59]. In these cases, CRL5 functions as an oncoprotein by targeting NOXA and DEPTOR for destruction. In contrast, active Src kinase promotes cancer cell invasion, proliferation, and survival by participating in various signaling pathways [60]. CRL5 specifically degrades the active Src protein and its tyrosine-phosphorylated targets, which prevent cell transformation and Src-induced cancer [61]. This demonstrates the tumor-inhibiting activity of CRL5. The studies discussed above illustrate that CRL5 activation has context-dependent roles in both tumor promotion and inhibition. However, CULLIN5 has a strong tumor-causing potential, and blocking its connection with RBX2 may present new opportunities for developing novel neddylation inhibitors for selective cancer treatment.

##### UBE2M with RNF111 and RNF 168 interactions

During DNA damage, NEDD8 accumulates at damage sites, requiring the E2 enzyme UBE2M and the E3 ubiquitin ligase RNF111 [62]. RNF111 interacts with UBE2M and enhances the polyneddylation of histone H4, which promotes the development of RNF168 foci and DNA repair. Knockdown of both UBE2M and RNF111 reduces RNF168 activity and hampers DNA repair. Additionally, NEDD8 can bind to histone 2 A (H2A) and prevent it from being ubiquitinated and degraded [63]. RNF168 promotes both H2A ubiquitination and neddylation while being itself a substrate for NEDD8. The neddylation of RNF168 is required for its E3 ubiquitin activity, and the knockdown of UBE2M decreases RNF168’s ligase activity. These findings indicate that UBE2M, RNF111, and RNF168 work together to repair DNA damage. Limiting their interactions may prevent DNA damage repair and induce apoptosis.

##### UBE2M-MDM2 interaction

MDM2 is a well-known oncoprotein that regulates the tumor suppressor protein p53 by functioning as a ubiquitin E3 ligase [64]. However, recent studies have shown that MDM2 can also act as a NEDD8 E3 ligase after being phosphorylated by c-Src. Once phosphorylated, MDM2 recruits UBE2M to inactivate p53 by neddylation, making the inhibition of UBE2M-MDM2 interactions a promising strategy for cancer treatment in wild-type p53 cells [65].

##### UBE2M-IAP interaction

The inhibitors of apoptosis proteins (IAPs) are endogenous inhibitors for programmed cell death. They contain two conserved regions- the baculovirus IAP Repeats (BIRs) and the really interesting new gene (RING) domains - that regulate caspases. The BIRs control the binding of IAPs to caspases, while the RING domain functions as a ubiquitin E3 ligase, causing auto-ubiquitination of IAPs and caspases. Research has demonstrated that IAPs can inhibit apoptosis by neddylation and deactivate caspase proteins using UBE2M. This suggests that targeting the interaction between UBE2M and IAP could be a promising strategy for developing anti-cancer drugs[66].

##### UBE2M- c-Cbl interaction

The E3 ligase c-Cbl promotes the neddylation and polyubiquitination of proto-oncogene c-Src, leading to its degradation by the proteasome. This inhibits cell migration and survival by deactivating the PI3K-AKT pathway [67]. Additionally, c-Cbl inhibits leukemia development by neddylating and stabilizing the type II receptor (TβRII), which decreases the levels of transforming growth factor β (TGF-β) [68]. Moreover, the UBE2M and c-Cbl axis have been shown to promote the neddylation of the EGFR, leading to its sorting and degradation, which prevents the overactivation of EGFR in cancer cells [69]. These findings demonstrate that neddylation, which is regulated by the UBE2M-c-Cbl interaction, has a tumor-inhibitory effect.

##### UBE2M-TFB3 interaction

TFB3 is a component of TFIIH, a well-studied complex involved in nucleotide excision repair (NER) and transcriptional start. It was discovered that the yeast E2 conjugating enzyme UBC12 allowed TFB3 to neddylate yeast Rtt101 and cullin3 in vivo. The impact of this relationship on humans hasn’t yet been studied, though [70].

#### Interactions of E2 with HECT type E3 ligases

##### UBE2M-DCN1 interactions

DCN1, a scaffold-type E3 ligase, plays a role in regulating the neddylation pathway and carcinogenesis and is often disrupted in several squamous cell carcinomas (SCCs) [6]. By binding to the acetylated N terminus of UBC12 and the winged-helix B domain of CULLIN1, DCN1 facilitates the neddylation of CULLIN1. Additionally, the DCN1-UBC12 interaction can be disrupted to selectively modulate CULLIN3 [43]. Overexpressing DCN1 promotes cancer cell proliferation and metastasis, as shown by its high mRNA and protein expression levels in various cancers [71]. Blocking DCN1 and E2 enzymes could inhibit selective neddylation, such as CULLIN3 neddylation. The co-crystal structure of UBC12-DCN1 reveals that DCN1 has a specific binding cavity that accommodates UBC12’s N-terminus [39]. Several inhibitors have targeted this interaction, which leads to the accumulation of CRL3 substrate NRF2 [47]. The compounds in this series may have broader use in treating diseases linked to the overproduction of reactive oxygen species.

##### UBE2M-ITCH interaction

The discovery of the ITCH, E3 ubiquitin ligase, during genetic research on mouse coat color variations revealed that ITCH has protein-interacting WW domains for substrate recruitment and a HECT domain for ubiquitin transfer [72]. ITCH regulates a broad range of immunological responses, including T-cell activation and tolerance and T-helper cell development. Haiwen li et al. discovered that ITCH modifies JUNB by NEDD8 [73]. JUNB is a proto-oncogene that belongs to the AP-1 transcription factor family. To neddylate JUNB, ITCH binds to UBE2M and facilitates NEDD8 binding to the N-terminal site of JUNB. Neddylation attenuates the transcriptional activity of JUNB by increasing polyubiquitination and degradation [73]. In metastatic prostate cancer, the loss of JUNB resulted in increased cancer cell proliferation [74]. While research suggests that JUNB has both cell division-promoting and -inhibiting actions, its manifestation varies depending on the cell-cycle state and surrounding circumstances. Accordingly, the inhibition of JUNB neddylation and protection from degradation by blocking the UBE2M-ITCH interaction could be beneficial for certain cancer treatments. Nonetheless, further studies on various types of cancer are required to confirm this function.

##### UBE2M-SMURF1 interaction

SMAD-specific E3 ubiquitin protein ligase 1 (SMURF1) is another HECT-type E3 ligase that conducts auto-neddylation on several lysine residues by physically interacting with NEDD8 and UBE2M [75]. Neddylation increases the ubiquitin ligase activity of SMURF1, which promotes cancer cell proliferation, invasion, and increases tumor volume in a mice xenograft model. Meanwhile, SMURF1 autoubiquitination decreases when UBE2M is depleted. Elevated expression of NEDD8, SMURF1, NAE1, and UBE2M have been found to correlate with cancer development and poor prognosis [75, 76]. These findings suggest that selective inhibition of UBE2M-SMURF1 could potentially reduce the oncogenic activity of SMURF1.

## Inhibitors reported against E2-based protein-protein interaction

**Fig. 4:**
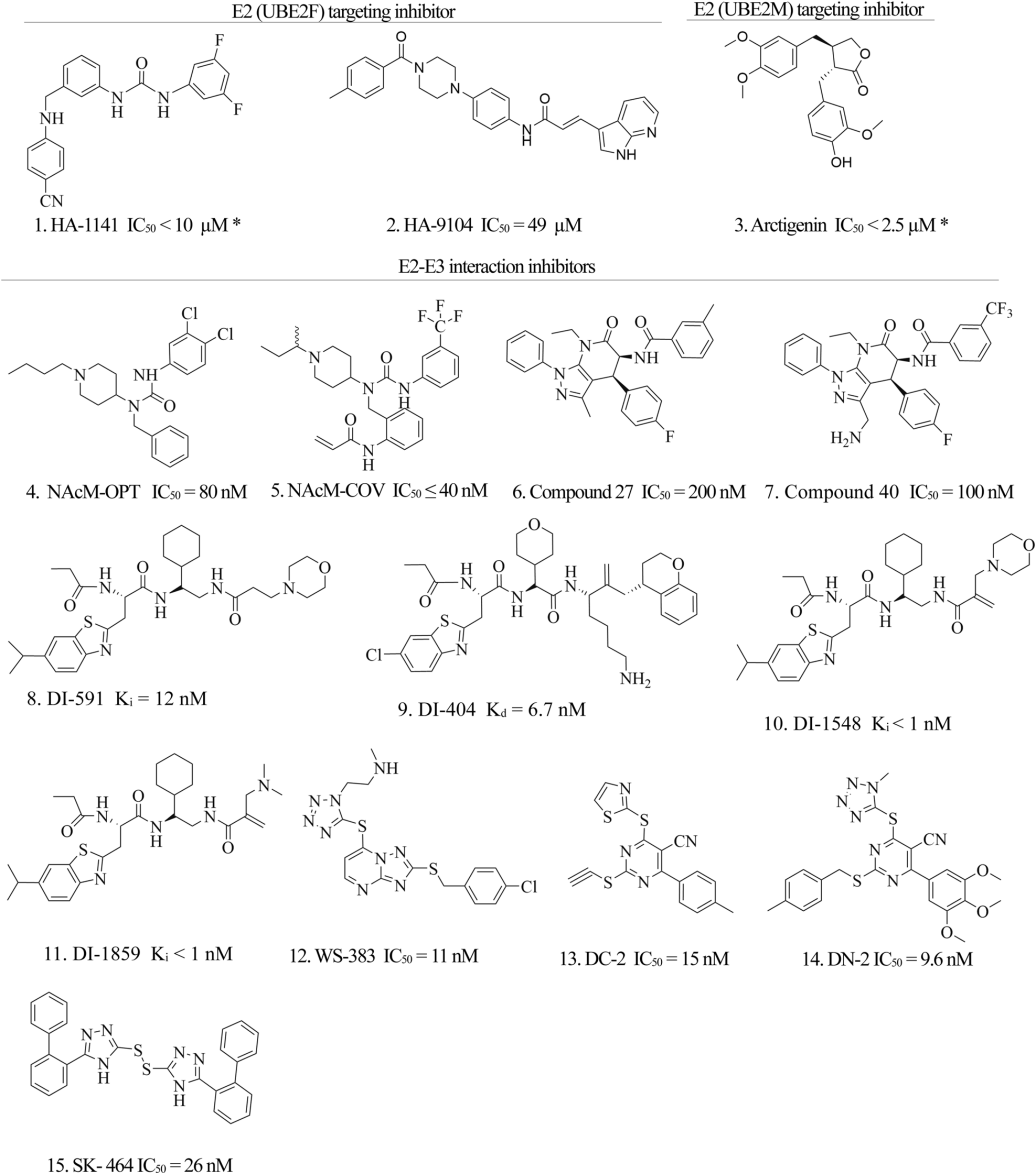
Inhibitors reported against various interaction sites of UBE2M. The picture represents the inhibitors reported regarding E2 interaction inhibition with E1 or E3, “*” indicate the IC_50_ measurement according to western blot band observation.

Currently, there are ongoing efforts to develop small molecules that target E2-based protein-protein interactions to inhibit neddylation. Several research groups have reported different types of inhibitors, as summarized in (Fig. 4 and Table 2). These include inhibitors targeting E2-E1 interactions and those targeting E2–E3 interactions, as reported in various studies.

**Table 2:** Inhibitors against E2-E1 or E2-E3 interactions.

Si No	Name & type	Potency	Target protein	Cellular Activity	References
1	HA-1141	IC_50_ < 10 µM^a^	UBA3	Inhibit CULLIN 1–5 neddylation and induce ER stress-dependent autophagy in H358 cells.	[38]
2	HA-9104	IC_50_ = 49 µM^a^	UBE2F	Inhibit CULLIN5 neddylation and accumulate CRL5 substrate NOXA	[77]
3	Arctigenin	IC_50_ < 2.5 µM	UBE2M	Inhibit CULLIN 1-4 neddylation and accumulate tumor suppressor PDCD4 protein.	[78]
4	NAcM-OPT	IC_50_ = 80 nM	DCN1	Inhibit CULLIN1 and CULLIN3 neddylation in 10 µM in HCC95 cells.	[79]
5	NAcM-COV	IC_50_ ≤ 40 nM	DCN1	Inhibit CULLIN1 and cullin3 neddylation at 10 µM in HCC95 cells.	[79]
6	Compound 27	IC_50_ = 0.2 µM	DCN1	Inhibit CULLIN1 and CULLIN3 neddylation at 10 µM in HCC95 cells	[80]
7	Compound 40	IC_50_ = 0.1 µM	DCN1	Thermally stabilizes DCN1, and inhibits anchorage-independent growth in a DCN1 amplified squamous cell carcinoma cell line	[81]
8	DI-591	K_i_ = 12 nM	DCN1	Inhibit CULLIN3 neddylation in 0.3 µM concentration in THLE2 cells.	[39]
9	DI-404	K_d_ = 6.7 nM	DCN1	Converts CULLIN3 into a predominantly unneddylated form at 1 μM in H2170 cells.	[82]
10	DI-1548	K_i_ < 1 nM	DCN1	Selectively inhibit CULLIN3 neddylation and accumulate NRF2 levels in U2OS cells.	[83]
11	DI-1859	K_i_ < 1 nM	DCN1	Selectively inhibit CULLIN3 neddylation and accumulate NRF2 levels in U2OS cells.	[83]
12	WS-383	IC_50_ = 11 nM	DCN1	Inhibit CULLIN1 and CULLIN3 neddylation in <3 µM in MGC-803 cells.	[84]
13	DC-2	IC_50_ = 15 nM	DCN1	Inhibit CULLIN1 and CULLIN3 neddylation at 10 µM in PC9 and H1975 cells.	[85]
14	DN-2	IC_50_ = 9.6 nM	DCN1	Inhibit CULLIN3 neddylation and reversed Ang-II induced cardiac fibroblast activation.	[86]
15	SK-464	IC_50_ = 26 nM	DCN1	Inhibit migration and invasion of cancer cells and CULLIN3 neddylation.	[87]

^a^Activity is reported based on the visual band of inhibition of CULLIN neddylation

### Targeting E2-E1 interaction site

Disrupting the docking of E2 in the E1 enzyme can inhibit selective E1-E2 neddylation interaction. E2 family proteins are characterized by numerous protein-protein interactions site, rigid conformations, and the absence of druggable pockets [77]. The small size and dynamic nature of the catalytic site of E2 enzymes make it challenging for small molecules to interact with them. However, recent studies have identified new inhibitors of UBE2M and UBE2F. Yanan Li et al. identified two pockets in UBE2F (F56 and V30) responsible for UBE2F-NAE interactions that are suitable for virtual drug screening (Fig. 2C). They demonstrated that HA-1141, a small molecule, can inhibit the collaboration between F56 and NAE [38]. Later, Tiantian Xu et al. described HA-9104, another small molecule capable of inhibiting the interaction between UBE2F and NAE when targeted to the V30 pocket [78]. In addition, Yi-fan Chen et al. conducted cell-based natural product inhibitor screening and observed UBE2M neddylation changes upon inhibitor application. They identified Arctigenin as a selective UBE2M inhibitor that inhibited UBE2M and CULLIN neddylation with an IC_50_ value of less than 2.5 μM [79]. These studies suggest that previously thought undruggable neddylation E2 enzymes can be targeted for the development of neddylation inhibitors.

### Targeting E2-E3 interactions

**Fig. 5:**
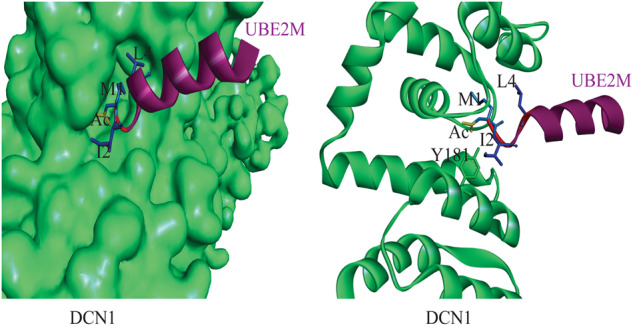
UBE2M and DCN1 interaction. Acetylated-UBE2M N-terminal (purple) binding in the pocket of DCN1 (left, green) and interacted amino acids, right (PDB:3TDU).

E2–E3 interactions play a critical role in substrate-specific neddylation, and although E2 can interact with various E3s for different substrate neddylation, it’s unclear how E2 chooses a specific E3 for specific substrate neddylation. However, studies have shown that the acetylation of UBE2M’s N-terminal site enhances its interaction with E3 ligase DCN1 (Fig. 5) [80]. The piperidine-based inhibitor NAcM-HIT targets the pocket of DCN1 and was identified with an IC_50_ value of 7 µM [81], followed by modifications that resulted in the identification of more potent and selective reversible DCN1 inhibitor NAcM-OPT with an IC_50_ value of 80 nM, and irreversible inhibitor NAcM-CoV with an IC_50_ value around 40 nM. However, piperidine-based inhibitors have some limitations, such as a minimal half-life and high dose necessity, making their clinical use challenging. An entirely new class of pyrazole-pyridone-based DCN1 inhibitors has been developed, with the primary compound showing 50% inhibition at 5.1 µM [82]. Subsequent modifications resulted in the identification of compound 27 with an improved inhibitory concentration of 200 nM. After improving the oral bioavailability of compound 27, a new inhibitor (compound 40) with two times more potency (IC_50_ 100 nM) was identified. DI591, a peptidomimetic small molecule with high affinity and cell-permeable, was developed by Haibin Zhou et al. from scratch against UBE2M-DCN1 interaction [39]. This compound showed a higher affinity to DCN1 with Ki 12 nM, and later, the same group reported another peptidomimetic small molecule, DI404, with a Kd value of 6.7 nM [83]. The most potent DCN1 inhibitors developed by the same group are DI-1548 and DI-1859, which showed 1000 times more potency in inhibiting CULLIN3 neddylation than DI-591 [84]. Four small molecules were reported as potential inhibitors of DCN1 by two different groups from Zhengzhou University. First, Shuai Wang et al. identified a triazolo [1,5-a] pyrimidine-based DCN1 inhibitor WS-291 with an IC_50_ value of 5.82 μM [85]. Later, WS-383 was found to be an effective and selective inhibitor of DCN1-UBE2M interaction with an IC_50_ value of 11 nM. Another class of DCN1 inhibitor, 5‑Cyano-6-phenyl-pyrimidin-based derivative DC-2 (IC_50_ = 15 nM) was obtained from primary HIT compound DC-1 (IC_50_ = 1.2 μM) [86]. Further SAR studies using DC-2 yielded DN-2 as a more potent inhibitor (IC_50_ = 9.6 nM) [87]. The same research group later reported phenyltriazole thiol-based derivatives SK-464, another new group of DCN1 inhibitors with an IC_50_ value of 26 nM [88].

Although various classes of selective UBE2M-DCN1 inhibitors have been reported, targeting ring-type E3 ligases such as RBX1, RBX2, MDM2, etc. have not reported yet. Small-molecule inhibitors have been used in the past to target RING-type E3 ligases by binding to protein-protein interfaces. This approach has been successful in inhibiting interactions such as HIF1α/VHL, p53/MDM2, p53/MDM4, Skp1/Skp2, and others [89]. To date, no inhibitors targeting E2-ring E3-based neddylation have been reported. One possible reason for this is the absence of a crystal structure elucidating E2-ring E3 interactions, which highlights the need for further investigation in future studies.

## Methods for E2-based inhibitor screening

Several methods have been employed to develop new drugs that target specific molecules, with examples including structure-based drug design, computer-aided drug design, and high-throughput screening using fluorescence [90, 91]. Furthermore, the application of these methods has been successful in the development of drugs for a range of diseases, and their usefulness in identifying inhibitors of neddylation based on E2 is promising. Continued research into these methods could lead to the discovery of new and effective neddylation inhibitors, which could prove highly beneficial in the treatment of various diseases.

### In silico method

Computer-based virtual screening has become an increasingly popular in-silico technique due to its affordability and effectiveness [90]. The principle of in silico drug design technique involves the use of computational methods to predict and analyze the interactions between drug-like molecules and biological targets. This process typically involves the use of computer models and simulations to evaluate the potential binding of a given drug compound to its target. This virtual screening method was utilized to discover the majority of NAE inhibitors. Regarding E2-based inhibitors, one such application of this technique involved identifying HA-1141 as an inhibitor of the interaction between UBE2F and UBA3 [38]. These findings demonstrate the existence of druggable pockets in neddylation E2s that can be exploited in the development of neddylation inhibitors. For docking, structured-based virtual screening, and drug design to take place, a crystal structure of the target protein is essential. Therefore, obtaining crystal structure information for E2-E1 or E2-E3 will be crucial for further research on this topic. While this technique is effective in identifying potential neddylation inhibitors, it is imperative to undertake subsequent validation and SAR optimization studies across various levels including biochemistry, cell biology, and animal pharmacology. These preclinical studies are crucial in identifying lead compounds that can advance to clinical trials.

### High throughput screening (HTS)

#### TR-FRET method

**Fig. 6:**
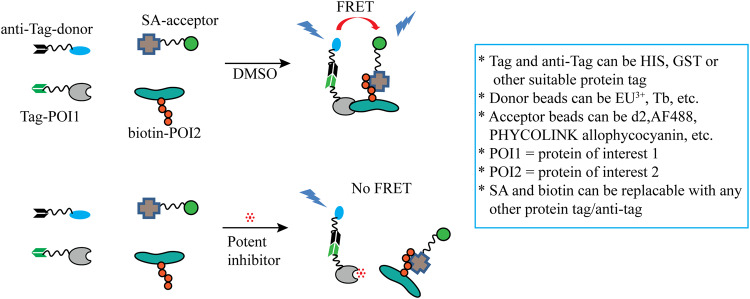
E2-based neddylation inhibitor discovery method. Schematic presentation of TR-FRET based HTRF method for drug discovery.

Time-resolved fluorescence resonance energy transfer (TR-FRET) is a technique used to examine complex molecular interactions in biochemical reactions using fluorescence. There are several methods of TR-FRET developed by different research groups. One common TR-FRET-based technique is homogeneous time-resolved fluorescence (HTRF) which is used to identify inhibitors of protein-protein interactions. The HTRF assay uses energy donor and acceptor molecules such as europium cryptate (Eu^3+^) and d2 respectively. In the proximity of these donor and acceptor molecules, FRET occurs, generating a signal (Fig. 6) [85]. Researchers have applied the principle of TR-FRET to identify inhibitors of neddylation using different optimization strategies. For example, a study by Wang Shuai et al. used antiGST-Eu^3+^ and streptavidin (SA)-d2 as donor and acceptor beads, respectively. The beads were able to bind with GST-DCN1 and biotin-conjugated N-terminal acetylated UBE2M. In the presence of DCN1 and Ac-N-UBE2M, the donor and acceptor beads came into proximity, generating a FRET signal. The signal was decreased upon successful inhibitor binding with DCN1. Similarly, a study used a TR-FRET assay with terbium (donor beads) and AF488 (acceptor beads) to identify DCN1 inhibitors, while another study used Eu^3+^ labeled Flag-M2-specific antibodies (as donor beads) and PHYCOLINK allophycocyanin-labeled GST-specific antibodies (as acceptor beads) to identify the NAE inhibitor MLN4924 [81]. In summary, targeted protein-protein interaction assays for inhibitor identification can be developed by selecting appropriate donor and acceptor beads, buffer solutions, and conjugated tag proteins.

#### AlphaScreen

Amplified luminescent proximity homogeneous assay (ALPHA), is a bead-based technology used for protein-protein interaction studies. Similar to the HTRF method, AlphaScreen is also based on the principle of energy transfer from a donor to an acceptor molecule during protein-protein interactions. In reaction, one of the interaction partners is conjugated to the donor beads, while the other partner is conjugated to the acceptor beads. Upon interaction of the two proteins, the donor and acceptor beads come into proximity, leading to energy transfer and luminescent signal generation. The AlphaScreen assay is highly sensitive, requires minimal sample, and can be used for high-throughput screening of large compound libraries. In the neddylation inhibitor study, the AlphaScreen was applied to identify Rhodium (III) complex as an NAE inhibitor.

#### SMM method

Small molecule microarray (SMM) enables the quick identification of small molecule-protein interactions. The SMM works based on a microarray made up of immobilized small molecules on a solid platform. These small molecules can include a wide range of compounds, including natural products, synthetic compounds, and drug-like molecules. The first step is to immobilize proteins of interest, such as enzymes or receptors, on the surface of the array, usually using a recombinant protein or an antibody probe. A small molecule library that has been fluorescently labeled is then added to the array. Following washing, the molecules that were bonded emit a fluorescent signal. Using a microarray scanner, it is possible to detect and measure the fluorescent signal of the molecule attached to the protein. The method was developed specifically to identify inhibitors for undruggable targets. The technique was employed to discover the UBC9 (an E2 conjugating enzyme involved in the sumoylation pathway) inhibitor [77] and it might potentially be useful for finding E2-based neddylation inhibitors.

#### Cell-based screening

During cell-based drug screening, the cells are typically pre-treated with the drug candidates for a defined period and then analyzed for a range of cellular responses along with protein expression. To identify an E2-based neddylation inhibitor, the method was applied by focusing on the neddylation status of E2 [79]. If a small molecule can successfully bind with the E2 protein, it may reduce the neddylation status of E2. However, there is also the possibility that the drug may bind to NAE and inhibit the neddylation of both NAE and E2. It is therefore important to simultaneously observe the neddylation status of both NAE and E2. To achieve selective E2 neddylation inhibition, the drug must be able to inhibit the neddylation of E2 while allowing the neddylation of NAE to continue unaffected.

## Conclusion

By modifying CULLIN proteins and other proteins unrelated to CULLIN, neddylation regulates their fate. Although just a few findings have revealed that neddylation inhibition accelerates the growth of cancer cells by reducing the cytotoxic effects of immune cells or by promoting glucose metabolism, a rising body of suggestion strongly recommends that neddylation reticence has anticancer properties through inducing apoptosis, autophagy, and senescence as well as suppressing angiogenesis, radiation, and chemotherapy resistance, and inflammatory responses [92–96]. As a result, Phase III trials are now underway for the potent and selective NAE inhibitor MLN4924 [97]. This drug has demonstrated excellent anticancer properties, especially when used in combination with chemotherapy. In some cases, MLN4924 encounters resistance or induces tumorigenesis but can be overcome by combination therapy [93, 98–100]. Although other NAE inhibitors have been developed, none have proven to be as efficient as MLN4924, except for TAS4464, which is in stage I/II clinical trials [101].

A significant part of the NEDD8 conjugation is determined by the E2 conjugating enzymes, which recruit selective E3 ligases. Keeping in touch with both E1 and E3 from the position of centrality, E2 maintains contact with both proteins. There is even an identical site on the surface of E2 that interacts with both E1 and E3 [16]. We discussed that UBE2M and UBE2F are accountable for the neddylation of CULLIN and other proteins in different ways. Despite UBE2M’s ability to neddylate most of the proteins, UBE2F neddylates only CULLIN5 [16]. Inhibiting both proteins genetically led to successful cancer treatment. Creating an artificial scarcity of UBE2M leaves tumor cells more vulnerable to chemotherapy, radiotherapy, DNA damage, and apoptosis. Similarly, UBE2F inactivation led to an increase in NOXA-dependent apoptosis and inhibited tumor growth. The E2s of neddylation also induce the immunosuppressive action of the tumor microenvironment, which disrupts cytotoxic T cell-mediated tumor eradication. In our discussion of E2, we have seen that E2 selectively binds with one E1 while interacting with more than ten E3. The inhibitors HA-1141, HA-9104, and arctigenin have already been found to inhibit E2-E1 interactions of the neddylation pathway. There is however a need for more intensive research to enhance the effectiveness of those inhibitors as anticancer drugs. We discussed that different E2-E3 interactions have different effects on cancer cells, depending on whether they are supportive or not. Hence, targeting selective E2-E3 interaction may lead to the discovery of selective anticancer drugs. As far as selective E2-based neddylation inhibitor discovery is concerned, the method mentioned here may prove useful.

In summary, the ongoing clinical studies for the NAE inhibitor MLN4924 demonstrate the promise of neddylation inhibition as an anticancer treatment. A developing issue is that MLN4924’s global neddylation inhibition can be optimized by targeting specific downstream neddylation. We have talked about how E2–E3 interactions are crucial for the selective neddylation of substrates. While some substrate neddylation inhibits cancer progression, other substrate neddylation supports cancer proliferation. In this situation, focusing on certain E2-E3 interactions may result in the development of novel anticancer drugs with high therapeutic potential.
